# New Energy Development and Pollution Emissions in China

**DOI:** 10.3390/ijerph16101764

**Published:** 2019-05-18

**Authors:** Ying Li, Yung-ho Chiu, Liang Chun Lu

**Affiliations:** 1 Business School, Sichuan University, Wangjiang Road No. 29, Chengdu 610064, China; liyinggs@scu.edu.cn; 2 Department of Economics, Soochow University, 56, Kueiyang St., Sec. 1, Taipei 10048, Taiwan; ryan@phanteks.com

**Keywords:** EBM (Epsilou-based measure), efficiency, new energy, traditional energy, undesirable output

## Abstract

China’s rapid economic growth is accompanied by increasing energy consumption and severe environmental problems. As sustainable development can only be achieved by reducing energy intensity, new energy and renewable energy investment, as well as improving traditional energy efficiency, is becoming increasingly important. However, past energy efficiency assessments using data envelopment analysis (DEA) models mostly focused on radial and non-radial DEA model analyses. However, traditional radial DEA models ignore non-radial slacks when evaluating efficiency values, and non-radial DEA models ignore the same proportionality as radial DEA when evaluating efficiency value slacks. To balance the radial and non-radial model characteristics and consider undesirable output, this study combines a modified Epsilou-based measure (EBM) DEA and undesirable output and proposes a modified undesirable EBM DEA model to analyze the efficiency of China’s new and traditional energy sources. The empirical results found that (1) most new energy investment in most municipalities/provinces rapidly grew from 2013 to 2016; (2) as the annual efficiency score was only 1 in Beijing, Inner Mongolia, Shanghai, and Tianjin, the other 26 municipalities/provinces need significant improvements; (3) traditional energy efficiency scores were higher than new energy efficiency; and (4) NO_2_ efficiencies are slightly better than CO_2_ and SO_2_ efficiencies.

## 1. Introduction

Energy drove the rapid economic growth in China, most of which was supplied from low-efficiency fossil energy sources. However, because fossil energy sources are limited and their use for power generation causes excessive carbon emissions that aggravate the greenhouse effect, the sustainable development of both natural and human environments is endangered. Therefore, in the past few decades, there was increased attention paid to new and renewable energies as a core alternative, as they are more environmentally friendly and sustainable than fossil fuels. Reducing energy use and improving energy efficiency by actively developing green and environmentally friendly new energy sources can guarantee a better life for future generations.

As China became the world’s leading energy consumer and the country that emits the highest carbon emissions, the Chinese government stated that, by 2030, the proportion of non-fossil fuels for energy consumption should rise to above 20%. China is also actively promoting a low-carbon economy that has high-efficiency and low-carbon emissions to improve the deteriorating environmental quality and ensure sustainable development. To reduce energy use and accelerate the generation of new energy, today’s new energy technologies are characterized by high performance, high efficiency, low cost, and low pollution.

Past energy research tended to focus more on energy efficiency than energy diversity [1,2,3,4,5,6,7,8,9,10,11,12]. However, in more recent years, there was a greater focus on the benefits of renewable energy and sustainable energy development [13,14,15,16,17,18,19,20,21,22,23,24,25,26,27,28] using data envelopment analysis (DEA) analysis models such as the radial CCR (Charnes, Cooper and Rhodes) and BCC (Banker, Charnes and Cooper) models or the non-radial slack-based model (SBM) or directional distance function (DDF) models. Unfortunately, because traditional radial DEA models ignore non-radial slacks and non-radial DEA models ignore the same proportionality as the radial DEA, they are not suitable for gaining a true picture of energy efficiency. To solve this problem, Tone and Tsutsui [29] suggested an Epsilou-based measure (EBM) variable range that was not limited and added an undesirable variable factor, which they called the modified undesirable SBM model. Therefore, this model was used in this paper to assess the energy efficiency of four Chinese municipalities and 26 provinces from 2013–2016 to avoid underestimating or overestimating the efficiency values and needed improvements.

Furthermore, although there were a number of new energy efficiency assessments suggested, there is a lack of general discussion about new energy and traditional energy efficiency. Therefore, to evaluate and analyze the environmental efficiency of new energy and traditional energy sources in four Chinese municipalities and 26 provinces from 2013–2016, this study used a modified undesirable EBM DEA model that had labor, fixed assets, new energy, and energy consumption as the input indicators, gross domestic product (GDP) as the output indicator, and CO_2_, SO_2_, and NO_2_ as the undesirable variable output indicators. 

The remainder of this paper is organized as the follows: Section 2 gives a comprehensive literature review, Section 3 describes the research method, Section 4 gives the comprehensive empirical results and discussion, and Section 5 gives the conclusions and policy proposals.

## 2. Literature Review

Data envelopment analysis (DEA) is a widely used linear programming technique. It evaluates the relative efficiency of a decision-making unit (DMU) based primarily on the concept of the Pareto optimal solution. DEA is an effective method to evaluate the priority of multiple decision-making schemes in a multi-oriented environment. Its main function is to establish an efficiency index by a set of evaluated decision-making units by measuring more than two attributes. This efficiency index forms the frontier of an efficiency boundary through the linear programming method by the input and output variable data of each DMU, and determines the relative efficiency of individual DMUs according to the distance between each DMU and the efficiency boundary. DEA uses a mathematical model to determine the production frontier. DEA differs from the stochastic frontier approach (SFA) in that it requires a preset production function. It is also different from multi-criteria decision analysis (MDA) when evaluating performance. The objectivity of weight is limited. DEA is considered to be more suitable for assessing company or industry performance than other methods (such as SFA) [30,31,32], and because of DEA requires very few assumptions and opened up possibilities for its use in many cases [33], the scope of DEA application was expanded to many industries. In recent years, DEA was widely used in energy efficiency [34,35,36,37,38].

As early research tended to be focused on environmental protection, it mainly discussed the impact of excessive greenhouse gas emissions on the global ecological environment, and any analyses were generally focused on energy efficiency. For example, Hu and Wang [1] used a modified radial DEA model to analyze China’s energy and found that economic growth boosted China’s energy efficiency. Yeh et al. [2] used a radial DEA model to analyze the energy efficiency of China and Taiwan, and found that Taiwan’s energy efficiency was higher than that of eastern China. Shi et al. [3] used a radial DEA model to analyze China’s energy efficiency, and found that energy efficiency in eastern China was the best. Choi et al. [4] used a slack-based DEA to analyze China’s energy efficiency, finding that China’s carbon dioxide efficiency was poor. Wu et al. [5] used radial DEA and Malmquist methods to explore energy efficiency in eastern, western, and central regions of China, and found that the average energy efficiency in eastern and central China was higher. In more recent research, Chang [6] used radial DEA to explore European Union (EU) energy efficiency and found that the main reason for the increase in energy intensity was whether the needed improvements were made. Wang and Wei [7] used a DDF model to analyze China’s energy efficiency and found that there was a significant growth in carbon dioxide emissions. Cui et al. [8] used radial DEA and Malmquist methods to analyze the relationship between management, technical indicators, and energy efficiency. Wu et al. [9] used a Russell measure model to explore China’s energy efficiency and found that excess energy was the main cause of poor energy efficiency. Pang et al. [10] used an SBM DEA to analyze the efficiency of 87 countries and found that European countries were more efficient in reducing emissions and had better energy efficiency. Guo et al. [11] also used SBM dynamic DEA to analyze the energy efficiency of 27 countries and found that all improved their energy efficiencies. Feng et al. [12] used a meta-frontier DEA to study the energy efficiency of 30 provinces in China and found that CO_2_ efficiency was generally low.

In addition to the above energy efficiency research, with the growth in pollution and carbon dioxide emissions, there was increased attention paid to new energy issues, renewable energy, and sustainable development. For example, Hoang and Rao [15] used a non-radial DEA to analyze the total efficiency of 29 OECD countries, and found that the sustainable efficiency varied enormously. Shiau and Jhang [16] used radial DEA to analyze the efficiency of Taiwan’s transportation system, and observed that, when the three core indicators (service impact, cost efficiency, and service reduction) were excellent, the transportation system could continue to develop. Camioto et al. [24] used an SBM DEA to analyze the overall efficiency of various industries in Brazil, finding that the textile industry was the most efficient industry in Brazil, and the metallurgical industry was the least efficient. Wang [25] used an SBM DEA to analyze the efficiency of 109 countries, finding that high-income countries performed best in terms of sustainable energy.

There are two major research directions for new energy issues: the impact of new energy on GDP or CO_2_, and new energy policy and efficiency assessments. Research on the impact of new energy on GDP or CO_2_ was mainly based on OLS (ordinary least square), VECM (vector error correction model), Panel, ECM (Error correction mechanism), ARDL (Autoregressive Distributed Lag), and VAR (vector autroregession) regression analyses [39,40,41,42,43,44,45,46,47]. Research also mainly explored new energy efficiencies and recommended the adoption of new energy policies. For example, Chien and Ho [13] used a radial DEA to analyze the total efficiency of 45 OECD (Economic Co-operation and Development) economies, and found that an increase in renewable energy improved technical efficiency. Honma and Hu [14] studied energy efficiency indicator structures in 47 metropolitan areas in Japan from 1993 to 2003, and found that renewable energy development was difficult to promote due to its excessive costs, which suggested that the government should encourage inefficient regions to change their industrial structures to reduce energy consumption. Blokhuis et al. [17] also used radial DEA to analyze the efficiency of new energy in the Netherlands and found that wind energy was able to improve technical efficiency. Boubaker [18] used radial DEA to analyze the energy efficiency of Morocco, Algeria, and Tunisia, and found that energy diversification was a common interest. Sueyoshi et al. [21] studied United States environmental efficiency and observed that a clean air act (CAA) was needed to improve carbon dioxide emissions. Fagiani et al. [20] explored the role played by renewable energy in power generation portfolios to reduce emissions in the power sector. Menegaki and Gurluk [19] compared renewable energy performances in Turkey and Greece, finding that Greece delayed its renewable energy development due to its economic crisis. Azande et al. [23] used fuzzy DEA to study Iranian wind power plants, concluding that consumer proximity was important to wind farm siting. Sueyoshi and Goto [22] used radial DEA to assess the efficacy of 160 photovoltaic power plants in Germany and the United States, finding that photovoltaic power plants in Germany were more efficient. Kim et al. [26] used a radial DEA method for an energy assessment and found that wind power was the most efficient renewable energy source for Korean government investment. Zhang and Xie [27] used a non-radial DDF method to explore renewable energy and sustainable development in China, concluding that China’s environmental supervision costs increased significantly from 1991 to 2005. Guo et al. [28] used a modified SBM model to explore energy savings and pollutant reductions in China, and came to the conclusion that the government needed to introduce new technologies to maintain economic development, and that all regions needed to pay attention to energy and pollution issues.

Therefore, while there were many previous papers that employed DEA for new energy efficiency assessments, there were few that jointly evaluated new energy and traditional energy efficiency. Furthermore, the main evaluation methods were radial or non-radial DEA models, both of which were shown to be prone to efficiency underestimations or overestimations. To solve this problem, in this paper, an undesirable variable factor was added to Tone and Tsutsui’s [29] EBM to propose a modified undesirable SBM Model to evaluate the energy efficiency of four municipalities and 26 provinces in China. 

## 3. Research Method

Based on Farrell’s [48] concept of “boundary” in data envelopment analysis, Charnes et al. [49] developed the CCR DEA model with a fixed-scale returns assumption, after which Banker et al. [50] extended these assumptions to propose a BCC model that measured technical efficiency (TE) and scale efficiency (SE). However, as both CCR and BCC were radial DEA models that ignore non-radial slacks when evaluating efficiency values, Tone [51] proposed a slack-based measure (SBM) in 2001 that used a difference variable as the basis for measurement, considering the slack in the input and output items and a scalar variable in the non-radial estimation methods to present SBM DMU (decision-making unit) efficiency values between 0 and 1, for which an efficiency value of 1 indicated that the DMU had no slack on the production boundary regardless of the input or output items. However, as the SBM was a non-radial DEA model, it failed to consider the radial characteristics; that is, it ignored the characteristics that had the same radial proportions. To address the shortcomings in both the radial and non-radial models, Tone and Tsutsui [29] then proposed the EBM (Epsilou-based measure) DEA model, that was input-oriented, output-oriented, and non-oriented, and was able to resolve the shortcomings in radial and non-radial DEA models.

Tone and Tsutsui’s [29] EBM DEA description for the input-oriented, output-oriented, and non-oriented model and solution is outlined below.

In the input-oriented EBM Model, the situation of resource inputs at the same output level is compared.

$$\gamma^{*}=\underset{0,\lambda ,S^{-}}{\min}\theta -\varepsilon_{x}\overset{m}{\underset{i=1}{\sum }}\frac{W_{i}^{-}S_{i}^{-}}{X_{io}}\;\mathrm{subject}\;\mathrm{to}\;\theta x_{0}-X\lambda -S^{-}=0,\;Y\lambda \geq y_{0},\;\lambda \geq 0,\;s^{-}\geq 0.$$

In the output-oriented EBM Model, the situation of output achievements at the same input level is compared.

$$1/\tau^{*}=\underset{\eta ,\lambda ,S^{+}}{\max}\eta +\varepsilon_{y}\overset{s}{\underset{i=1}{\sum }}\frac{W_{i}^{+}S_{i}^{+}}{y_{io}}\;\mathrm{subject}\;\mathrm{to}\;X\lambda \leq x_{0},\;\eta y_{0}-Y\lambda +S^{+}=0,\;\lambda \geq 0,\;s^{+}\geq 0.$$

### 3.1. Non-Oriented EBM: Simultaneous Assessment of Inefficiency from Both Input and Output Perspectives

Suppose there are n DMUs, $DMU_{j}=\left(DMU_{1},DMU_{2},\cdots ,DMU_{k},\cdots ,DMU_{n}\right)$, that have m type inputs $X_{j}=\left(X_{1j},X_{2j},\cdots ,X_{mj}\right)$, that produce an s type output $Y_{j}=\left(Y_{1j},Y_{2j},\cdots ,Y_{sj}\right)$; then, the efficiency of the DMU is:

(1) $$K^{*}=\underset{0,\eta ,\lambda ,s^{-},s^{+}}{\min}\frac{\theta -\varepsilon_{x}\sum_{i=1}^{m}\frac{w_{i}^{-}s_{i}^{-}}{x_{i0}}}{\eta +\varepsilon_{y}\sum_{i=1}^{s}\frac{w_{i}^{+}s_{i}^{+}}{y_{i0}}}\;\mathrm{Subject}\;\mathrm{to}\;\theta X_{0}-X_{\lambda }-S^{-}=0,\;\eta Y_{0}-Y_{\lambda }+S^{+}=0,\;\lambda_{1}+\lambda_{2}+\cdots +\lambda_{n}=1\;\lambda \geq 0,\;S^{-}\geq 0,\;S^{+}\geq 0.$$

where *Y* is the DMU output, *X* is the DMU input, $S^{-}$ is the slack variable, $S^{+}$ is the surplus variable, $W^{-}$ is the weight of input I, $\sum W_{i}^{-}=1\;\left(\forall_{i}W_{i}^{-}\geq 0\right)$, $W^{+}$ is the weight of output *S*, $\sum W_{i}^{+}=1\;\left(\forall_{i}W_{i}^{+}\geq 0\right)$, $\varepsilon_{x}$ is a combination of radial θ and non-radial slack, and $\varepsilon_{y}$ is a combination of radial η and non-radial slack.

If DMU0 $K^{*}=1$ is the best efficiency for a non-oriented EBM, then if an inefficient DMU wants to achieve an appropriate efficiency goal, the following adjustments are needed:

$$X_{0}^{*}=X\lambda^{*}=\theta^{*}X_{0}-S^{-*};\;Y_{0}^{*}=Y\lambda^{*}=\eta^{*}y_{0}+S^{+}.$$

### 3.2. Empirical Model in This Study: A Modified Undesirable EBM DEA Model

Because Tone and Tsutsui’s [29] EBM had no restrictions for the range of *θ* and *η* variables and did not consider any undesirable factors, this paper combines the modified EBM DEA and an undesirable factor for the evaluation of the energy efficiency of 30 mainland Chinese municipalities/provinces so as to avoid underestimating or overestimating the efficiency values.

In the modified undesirable EBM DEA model, the objective is to expand desirable outputs while simultaneously reducing inputs and undesirable output. The modified undesirable EBM DEA Model is described below.

Suppose there are n DMUs, $DMU_{j}=\left(DMU_{1},DMU_{2},\cdots ,DMU_{k},\cdots ,DMU_{n}\right)$, using m type inputs $X_{j}=\left(X_{1j},X_{2j},\cdots ,X_{mj}\right)$ and producing s type outputs $Y_{j}=\left(Y_{1j},Y_{2j},\cdots ,Y_{sj}\right)$; then, the DMU efficiency is as follows

(2) $$K^{*}=\underset{0\eta ,\lambda ,s^{-},s^{+g},s^{-b}}{\min}\frac{\theta -\varepsilon_{x}\sum_{i=1}^{m}\frac{w_{i}^{-}s_{i}^{-}}{x_{i0}}}{\eta +\varepsilon_{y}[\sum_{i=1}^{S1}\frac{w_{i}^{+S1}s_{i}^{+g}}{y_{i0}}+\sum_{i=1}^{S2}\frac{w_{i}^{-S2}s_{i}^{-b}}{y_{i0}}]}\;\mathrm{Subject}\;\mathrm{to}\;\theta X_{0}-X_{\lambda }-S^{-}=0,\;\eta Y_{0}-Y_{\lambda }^{+g}+S^{+g}=0\;\eta Y_{0}-Y_{\lambda }^{-b}+S^{-b}=0\;\lambda_{1}+\lambda_{2}+\ldots +\lambda_{n}=1\;\;\lambda \geq 0,\;S^{-}\geq 0,\;S^{+g}\geq 0,\;S^{-b}\geq 0,\;\theta \leq 1,\;\eta \geq 1$$

where *Y* is the DMU output, *X* is the DMU input, $S^{-}$ is the slack variable, $S^{+g}$ is the desirable slack variable, $S^{-b}$ is the undesirable slack variable, $W^{-}$ is the weight of input i, $\sum W_{i}^{-}=1\;\left(\forall_{i}\;W_{i}^{-}\geq 0\right)$, $W^{+}$ is the weight of output *S*, $\sum^{\;}W_{i}^{+S1}+\sum^{\;}W_{i}^{-S2}=1\;\left(\forall_{i}\;W_{i}^{+}\geq 0\right),\;\varepsilon_{x}$ is the combination of radial θ and non-radial slack, and $\varepsilon_{y}$ is the combination of radial η and non-radial slack.

If $DMU_{0}$ $K^{*}$ = 1 is the best efficiency for the non-oriented EBM, then an inefficient DMU needs the following adjustments to achieve the most appropriate efficiency goal:

$$X_{0}^{*}=X\lambda^{*}=\theta^{*}X_{0}-S^{-*};\;Y_{0}^{*}=Y\lambda^{*\left(-b\right)}=\eta^{*}y_{0}+S^{+g};\;Y_{0}^{*}=Y^{*-b}\lambda =\eta^{*}y_{0}+S^{-b}.$$

### 3.3. New Energy, Energy Consumption, and CO_2_, SO_2_, and NO_2_ Efficiency Indices

Hu and Wang’s [1]’s total-factor energy efficiency index is used in this paper to overcome any possible bias in the traditional energy efficiency indicators. For each specific evaluated municipality or province, the GDP, energy consumption (ENG), new energy (NENG), and CO_2_, SO_2_, and NO_2_ efficiencies were calculated using Equations (3)–(8).

(3) $$\mathrm{GDP}=\frac{\mathrm{Actual}\;\mathrm{GDP}\;\mathrm{desirable}\;\mathrm{output}\;\left(i,t\right)}{\mathrm{Target}\;\mathrm{GDP}\;\mathrm{desirable}\;\mathrm{output}\;\left(i,t\right)};$$

(4) $$\mathrm{ENG}=\frac{\mathrm{Target}\;\mathrm{energy}\;\mathrm{input}\;\left(i,t\right)}{\mathrm{Actual}\;\mathrm{energy}\;\mathrm{input}\;\left(i,t\right)};$$

(5) $$\mathrm{NENG}=\frac{\mathrm{Target}\;\mathrm{new}\;\mathrm{energy}\;\mathrm{input}\;\left(i,t\right)}{\mathrm{Actual}\;\mathrm{new}\;\mathrm{energy}\;\mathrm{input}\;\left(i,t\right)};$$

(6) $${\mathrm{CO}}_{2}=\frac{{\mathrm{Target}\;\mathrm{CO}}_{2}\;\mathrm{Undesirable}\;\mathrm{output}\;\left(i,t\right)}{{\mathrm{Actual}\;\mathrm{CO}}_{2}\;\mathrm{Undesirable}\;\mathrm{output}\;\left(i,t\right)};$$

(7) $${\mathrm{SO}}_{2}=\frac{{\mathrm{Target}\;\mathrm{SO}}_{2}\;\mathrm{Undesirable}\;\mathrm{output}\;\left(i,t\right)}{{\mathrm{Actual}\;\mathrm{SO}}_{2}\;\mathrm{Undesirable}\;\mathrm{output}\;\left(i,t\right)};$$

(8) $${\mathrm{NO}}_{2}=\frac{{\mathrm{Target}\;\mathrm{NO}}_{2}\;\mathrm{Undesirable}\;\mathrm{output}\;\left(i,t\right)}{{\mathrm{Actual}\;\mathrm{NO}}_{2}\;\mathrm{Undesirable}\;\mathrm{output}\;\left(i,t\right)}.$$

If the target ENG and NENG input are equal to the actual input and the CO_2_, SO_2_, and NO_2_ are equal to the actual undesirable outputs, then the ENG, NENG, and CO_2_, SO_2_, and NO_2_ efficiencies are equal to 1, indicating overall efficiency. If the target ENG and NENG input is less than the actual input and the CO_2_, SO_2_, and NO_2_ undesirable outputs are less than the actual undesirable outputs, then the ENG, NENG, and CO_2_, SO_2_, and NO_2_ efficiencies are less than 1, indicating overall inefficiency.

If the target GDP desirable output is equal to the actual GDP desirable output, then the GDP efficiency is equal to 1, indicating overall efficiency. If the actual GDP desirable output is less than the target GDP desirable output, then the GDP efficiency is less than 1, indicating overall inefficiency.

## 4. Empirical Analyses

### 4.1. Data Sources and Description

This study used 2013 to 2016 panel data from 30 Chinese municipalities/provinces in the most developed areas in China. The socio-economic development data were collected from the Chinese Statistical Yearbooks [52], the Demographics and Employment Statistical Yearbook of China, and the City Statistical Yearbooks [53]. Air pollutant data were collected from the Chinese Environmental and Protection Bureau Annual Reports and the Chinese Environmental Statistical Yearbooks [54].

As the 30 municipalities/provinces have different populations, industries, natural resources, meteorological conditions, and geographical positions, they were fairly representative of the pollution emissions and treatment situations in China.

The input indicator variables used in this study were labor, fixed assets, new energy, and traditional energy consumption, the output indicator was GDP, and CO_2_, SO_2_, and NO_2_ were the undesirable output (Table 1).

#### 4.1.1. Input Variables

Labor input (lab): this study used the number of employees in each municipality/province at the end of each year (unit = people).

Capital input (assets): the capital stock was calculated based on the fixed asset investments in each municipality/province (unit = 100 million Chinese yuan (CNY)).

Energy consumption (com): this was calculated from the total energy consumption in each municipality/province (unit = 100 million tons).

New energy (new). In October 2012, the State Council issued “China’s energy policy 2012” Chapter 4 [55], developing new and renewable energy, in which nuclear energy is a key project for the development of new energy in the country, aiming to optimize the energy structure and ensure national energy security. Due to the nuclear disaster caused by the 2011 earthquake in Japan, it is still controversial whether countries can summarize nuclear energy into green energy.

For China’s development, because of the continuous improvement of science and technology, new energy generally refers to the development of new technologies including hydropower, wind power, solar energy, biomass energy, nuclear energy, geothermal energy, wave energy, ocean current energy, tidal energy, and combustible ice. Microbial energy, hydrogen energy, and fourth-generation nuclear energy are all important projects for China’s future energy development.

Thus, new energy included solar energy, nuclear energy, and wind power. It was calculated from the total energy consumption in each municipality/province (unit = 100 million tons).

#### 4.1.2. Output Variable

GDP: the GDP in each municipality/province was applied as the output (unit = 100 million CNY). The GDP data were extracted from each province’s statistical yearbook for the given period.

#### 4.1.3. Undesirable Output

The CO_2_ (carbon dioxide) emissions data for each municipality/province were estimated from the energy consumption. CO_2_ emissions are a primary cause for the changes being experienced in earth temperatures and the rising sea levels. CO_2_, unlike other air pollutants, is used as the sole carbon emissions measure for global solutions to climate change. SO_2_ (sulfur dioxide), which is released naturally by volcanic activity, is also a by-product from the burning of fossil fuels contaminated with sulfur compounds. NO_2_ (nitrogen dioxide), which is from a group of highly reactive gases known as nitrogen oxides (N_X_), is an intermediate gas resulting from the industrial synthesis of nitric acid, millions of tons of which are produced each year. At higher temperatures, it is a reddish-brown gas that has a characteristic sharp, biting odor and is one of the most prominent air pollutants.

### 4.2. Statistical Analysis

**Figure 1:**
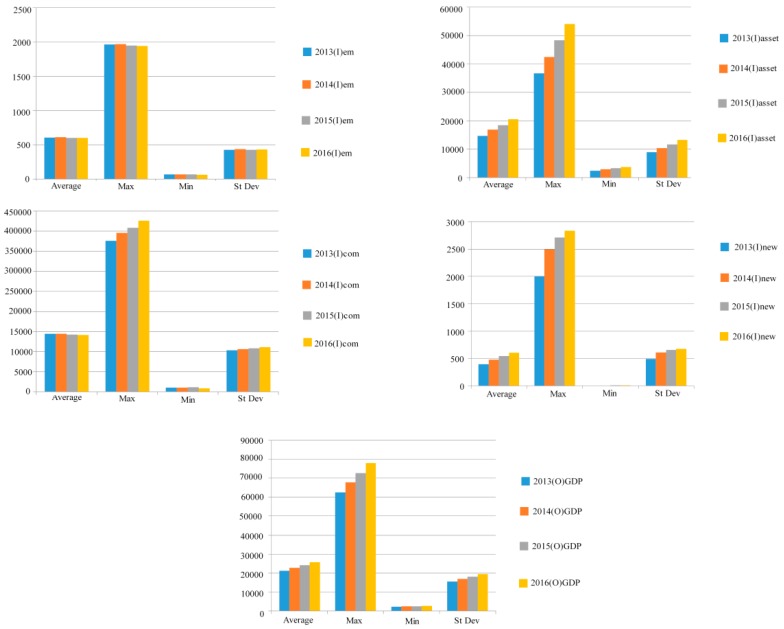
Input and output indicators. Sources: the Chinese Statistical Yearbooks [52], the Demographics and Employment Statistical Yearbook of China, and the City Statistical Yearbooks [53]. Air pollutant data were collected from the Chinese Environmental and Protection Bureau Annual Reports and the Chinese Environmental Statistical Yearbooks [54].

Figure 1 shows the statistical analyses for the employed population, fixed assets, traditional energy consumption inputs, new energy production inputs, and GDP. From the statistical analysis, it can be seen that the maximum and average number of employed people declined from 2014, and investment in fixed assets rose significantly. The average GDP maintained a steady upward trend, where the maximum GDP had a relatively large upward trend, and minimum GDP slowly increased. Although the average value of traditional energy sources continued to decline slightly, the maximum traditional energy consumption continued to rise, and the minimum value experienced only a marginal rise. The total new energy production was smaller than the traditional energy consumption; however, from 2012 to 2013, there was a significant increase from 2000 tons to 2500 tons with a further rise from 2013 to 2016. Therefore, it can be seen from the rapid growth that, under central government guidance, most municipalities and provinces were seriously investing in new energy.

### 4.3. Empirical Analysis of the Modified Undesirable EBM DEA

This study used a modified Undesirable EBM DEA model to analyze the energy efficiencies in 30 Chinese municipalities/provinces.

#### 4.3.1. Epsilon Score Analysis

The sample Epsilon score in this study compared the radial DEA and the non-radial DEA. The main radial analysis was close to 0 and the main non-radial analysis was close to 1. Table 2 indicates that the radial DEA model was more appropriate for this analysis.

#### 4.3.2. Annual Efficiency

Table 3 shows the total efficiency scores for the four municipalities (Beijing, Shanghai, Tianjin, and Chongqing) and the 26 provinces from 2013 to 2016. It can be seen that total efficiencies of 1 were achieved by Beijing, Inner Mongolia, Shanghai, and Tianjin, with the other municipalities/provinces having relatively high total annual efficiencies. The areas with a full four-year efficiency score below 0.6 include Gansu, Guizhou, Xinjiang, and Yunnan, where Gansu is the worst of the 30 cities with an efficiency score below 0.5 in the full four years, and the efficiency score continually dropped to 0.41 in 2016, suggesting very large room of improvement. In addition to the four regions with an efficiency score of 1, the four-year efficiency scores of the other 26 regions showed different trends. It can be seen that only four regions had a total efficiency score that continued to rise or fluctuate including Guizhou, Heilongjiang, Liaoning, and Sichuan. The biggest increase was in Liaoning, rising from 0.74 in 2013 to 1 in 2016, and the efficiency improvement was significant. The overall efficiency scores of the other 22 regions continued to decline or fluctuate. In most areas, the decline was less than 0.1. Hebei had the highest decline, from 0.79 in 2013 to 0.66 in 2016.

#### 4.3.3. Comparison of Radial and Non-Radial Inefficiency Analysis for the Input and Output Indicators

Table 4 shows the 2013 input indicator inefficiency scores, radial inefficiency scores, and non-radial inefficiency scores. Gansu (0.35), Guizhou (0.31), Shanxi (0.31), Xinjiang (0.32), and Heilongjiang, Henan, Ningxia, and Qinghai provinces with inefficiencies of around 0.25 had the highest input indicator inefficiencies, followed by Chongqing and Jiangxi at around 0.2, with most other municipalities/provinces being between 0.1 and 0.2. From the comparison of the input indicator radial inefficiency and the non-radial inefficiency scores, it can be seen that most municipalities/provinces had higher radial inefficiency scores than non-radial inefficiency scores. However, the input indicator non-radial inefficiency scores in seven provinces (Fujian, Guangdong, Hainan, Hebei, Jiangsu, Shandong, and Zhejiang) were still slightly higher than the radial inefficiency scores. The input indicator inefficiency scores were mainly caused by the non-radial inefficiency scores, or were partly caused by the radial inefficiency scores and partly caused by the non-radial inefficiency scores.

The output indicator inefficiency scores, the radial inefficiency scores, and the non-radial inefficiency scores were above 0.2 in nine provinces or municipalities: Gansu, Guizhou, Heilongjiang, Henan, Qinghai, Shanxi, Shaanxi, Xinjiang, and Yunnan. Moreover, the radial inefficiency scores were higher than the non-radial inefficiency scores, which indicated that the output inefficiency scores in these nine provinces were mainly caused by radial inefficiencies. Other municipalities/provinces had inefficiencies ranging from 0 to 0.2, with all scores being dominated by the radial inefficiency scores, as the non-radial inefficiency scores were smaller. Table 5 shows that there were only four provinces/municipalities with an inefficiency score of 0. These four regions are Beijing, Inner Mongolia, Shanghai, and Tianjin. There were four regions with the highest inefficiency scores, all with scores above 0.3, including Gansu, Guizhou, Shanxi, and Xinjiang. The lowest inefficiency score was in Guangdong, only about 0.11; the second lowest was in Shandong, with an inefficiency score around 0.13. In addition to the lowest inefficiency scores in the above two regions, there were other 11 regions with an inefficiency score below 0.2. The inefficiency scores for the remaining 13 regions ranged from 0.2 to 0.3. The data in the table analyzed the inefficiency scores for each region and were affected by radial and non-radial inefficiency scores. The output inefficiency scores of all regions were mainly affected by the radial inefficiency score.

Table 6 shows that, in 2015, only Beijing, Inner Mongolia, Shanghai, and Tianjin had inefficiency scores of 0. The radial and non-radial inefficiency scores for the remaining 26 municipalities/provinces were generally higher than in 2014, with the inefficiency score for the Gansu input index being 0.4 or higher, followed by Shanxi and Xinjiang with an increase of 0.37. The inefficiency score in Shaanxi was 0.29, while that in Yunnan and Ningxia was 0.28, that in Qinghai was 0.27, and that in Guizhou and Henan was around 0.22. The input indicator inefficiency scores in the other nine municipalities/provinces were between 0.1 and 0.2. Gansu, Shanxi, and Xinjiang provinces had output indicator inefficiency scores above 0.3, whereas the output indicator inefficiency scores in Guizhou, Henan, Jiangxi, Ningxia, Qinghai, Shaanxi, and Yunnan ranged from 0.3 to 0.2. Anhui, Chongqing, Guangxi, Hebei, Heilongjiang, Hubei, Jilin, and Sichuan had inefficiency scores of between 0.1 to 0.2, and Fujian, Guangdong, Hainan, Hunan, Liaoning, Shandong, and Zhejiang had output indicator inefficiency scores from 0 to 0.1. Only Hainan, Hebei, Hunan, and Jiangsu were mainly affected by the non-radial inefficiency scores, with the scores in the other municipalities/provinces being mainly caused by the radial inefficiency scores. The output indicators indicated that, except for Liaoning, the output inefficiency in the other municipalities/provinces was mainly because of the radial inefficiency scores.

Table 7 shows that, in 2016, only Beijing, Liaoning, Inner Mongolia, Shanghai, and Tianjin had inefficiency scores of 0. The radial and non-radial inefficiency scores for the remaining 25 municipalities/provinces were generally higher than in 2015. The input indicator inefficiency scores in Xinjiang, Shaanxi, Shanxi, Ningxia, Guizhou, and Gansu were above 0.3, and those in Yunnan, Qinghai, Jilin, Jiangxi, Henan, Heilongjiang, Hebei, Chongqing, and Anhui were between 0.2 and 0.3. Some municipalities/provinces had output indicator inefficiency scores exceeding 0.2, with Xinjiang, Shaanxi, and Gansu having output indicator inefficiency scores higher than 0.3. Except for Hainan, Hunan, Jiangsu, and Shandong, which were affected by the non-radial inefficiency scores, the input indicator inefficiency scores in most municipalities/provinces were generated from the radial inefficiency scores. Only the output indicator inefficiency scores in Shandong were mainly affected by the non-radial inefficiency scores, with all other municipalities/provinces being mainly affected by the non-radial efficiency scores.

#### 4.3.4. Efficiency of the Input and Output Indicators: Fixed Assets, Employees, GDP, Energy, New Energy, and CO_2_, SO_2_, and NO_2_

Table 8 shows the fixed asset and employment efficiencies in the municipalities/provinces from 2013 to 2016. As can be seen, there were significant fluctuations in the input indicator efficiencies across the municipalities/provinces. All municipalities/provinces had large fixed asset efficiency fluctuations, with only Beijing, Inner Mongolia, Shanghai, and Tianjin achieving fixed asset efficiency scores of 1. All other municipalities/provinces need significant improvement. The areas where the efficiency scores of fixed assets continued to rise or fluctuate include Guangdong, Guizhou, Hainan, Heilongjiang, Liaoning, Shandong, Sichuan, and Zhejiang. The efficiency scores of fixed assets in the other 18 regions showed sustained or fluctuating decline.

The employment efficiency scores in all municipalities/provinces were higher, with those in Beijing, Inner Mongolia, Shanghai, and Tianjin achieving 1, while all others scored above 0.6. In most regions, this indicator fluctuated or continued to decline.

Table 9 shows the new energy, traditional energy consumption, and GDP efficiency scores in the municipalities/provinces. From the traditional energy consumption efficiency score, it can be seen that only the efficiency scores of Beijing, Inner Mongolia, Shanghai, and Tianjin were all 1. There is significant room for improvement in the efficiency of this indicator in other regions. The areas with a four-year efficiency score below or equal to 0.5 include Anhui, Gansu, Hebei, Heilongjiang, Jilin, Ningxia, Shanxi, Shaanxi, and Xinjiang. Among them, the least efficient was Shanxi, as its four-year efficiency score was only about 0.14, suggesting much room for improvement. It can be seen from the changes that the annual difference in the efficiency scores of each region was also large and presented different trends. Only seven regions, such as Guangxi, Guizhou, Liaoning, Qinghai, Sichuan, and Yunnan, had scores that fluctuated or continued to rise. The efficiency scores of the other 19 regions fluctuated or continued to decline.

Compared with the traditional energy and other indicator efficiencies, the new energy efficiencies were generally very low, and had obvious fluctuations. In addition to Beijing, Inner Mongolia, Shanghai, and Tianjin had new energy efficiencies of 1 for four consecutive years, whereas most other areas have much room for improvement. Only Henan’s new energy efficiency scores ranged from 0.11 to 0.15 for all four years. Anhui, Jiangsu, and Shandong had a new energy efficiency score of 0.1 to 0.2 for one or two years.

The other 23 regions had new energy efficiency scores below 0.1, which suggests great room for improvement. New energy efficiency scores in all 26 regions suggest room for improvement, as they continued to decline or fluctuated.

The GDP efficiency scores in all municipalities/provinces were high, with most being over 0.8. However, there were few fluctuations and the efficiencies generally remained around the same or slightly declined over the four years, with only Guizou, Heilongjiang, Hunan, and Liaoning showing a small increase in efficiency and volatility, whereas the efficiency of the other 22 regions continued to decline or fluctuated slightly.

Table 10 shows the 2013–2016 CO_2_, SO_2_, and NO_2_ efficiency scores in the municipalities/provinces.

The CO_2_ efficiencies varied significantly across the municipalities/provinces. The CO_2_ efficiency scores in Beijing, Inner Mongolia, Shanghai, and Tianjin were 1 for all four years, and the CO_2_ efficiency scores in other regions varied widely. For example, Anhui, Gansu, Guizhou, Hebei, Heilongjiang, Ningxia, Shanxi, Shaanxi, and Xinjiang all had scores lower than 0.4. Among them, Ningxia and Shanxi’s CO_2_ emission efficiency was very poor for all four years, with the highest only being 0.13 and 0.14, suggesting much room for improvement. Only the efficiency scores of seven regions including Guizhou, Qinghai, Sichuan, and Yunnan showed a small fluctuation or continued increase. The efficiency scores of the other 19 regions fluctuated or continued to decline, and the decline was significant.

The SO_2_ efficiencies in all municipalities/provinces were slightly higher than the CO_2_ efficiencies; however, there were large differences. While the SO_2_ efficiency scores in Beijing, Inner Mongolia, Shanghai, and Tianjin were 1, in the other municipalities/provinces, they tended to fluctuate over time. The worst performance was in Ningxia, which had an efficiency score of only 0.11 and below for all four years, followed by Shanxi and Xinjiang, both of which had a four-year efficiency score of less than 0.2 with a lot of room for improvement. There are five regions where all efficiency scores fluctuated or continued to rise, including Chongqing, Guangdong, Guizhou, Liaoning, and Sichuan. The largest increase was in Liaoning, rising from 0.44 in 2013 to 1 in 2016. The efficiency scores of the other 20 provinces/municipalities fluctuated or continued to decline. The largest decline was in Anhui, which fell from 0.64 in 2013 to 0.56 in 2016.

The NO_2_ efficiencies were relatively higher than the SO_2_ in most municipalities/provinces; however, there were also large differences. While the NO_2_ efficiencies in Beijing, Inner Mongolia, Shanghai, and Tianjin were 1 across all years, the NO_2_ efficiencies in the other municipalities/provinces fluctuated over time. Ningxia had the lowest efficiency for all four years, with the highest score being only 0.15 in 2013. Shanxi and Xinjiang followed, whereby all of its four-year efficiency scores were below 0.28. Gansu and Hebei’s four-year efficiency score was lower than 0.4, suggesting room for improvement. The efficiency scores of various regions also showed a large trend, but there were only five regions that fluctuated or continued to rise, including Guangdong, Guangxi, Guizhou, Henan, and Liaoning. The biggest increase was still in Liaoning, rising from 0.67 in 2013 to 1 in 2016. The other 21 regions experienced a small sustained or volatile decline.

The overall pollution analysis and the rankings for the different pollutants in each municipality/province are shown in Table 11.

## 5. Conclusions and Policy Implications

The rapid economic growth in China led to a significant rise in energy consumption, which in turn led to a rise in pollutant emissions and environmental problems, thereby threatening China’s sustainable development goals. Therefore, China needs to reduce its energy intensity through investment in new energy and renewable energy. This study proposed a modified undesirable EBM DEA model to analyze new and traditional energy efficiencies in 30 municipalities and provinces, the conclusions from which are given below.

1. The comparison of the input and output indicator radial DEA and non-radial DEA inefficiency scores found that most input indicator inefficiencies were due to the radial DEA, with only a few municipalities/provinces having inefficiencies resulting from the non-radial DEA.
2. The annual efficiency was 1 in Beijing, Inner Mongolia, Shanghai, and Tianjin for all four years from 2013–2016. The other 26 municipalities/provinces had large differences and required significant improvements. The annual total efficiency score changes in most municipalities/provinces had variable trends.
3. The various input and output indicator efficiencies for employment, GDP, and fixed assets were generally higher. However, the traditional energy efficiency scores and new energy efficiency scores were generally low, with the new energy efficiency scores being lower than the traditional energy efficiency scores.
4. The CO_2_, SO_2_, and NO_2_ efficiency scores varied widely, with the NO_2_ efficiencies being slightly better than the CO_2_ and SO_2_ efficiencies. However, the efficiency scores for these three undesirable outputs varied considerably across the municipalities/provinces.

### Policy Implications

1. Except for the municipalities/provinces that had efficiency scores of 1, only two or three provinces had overall upward efficiency trends; however, the overall annual efficiency in most other provinces declined, indicating that more effective measures are needed to improve the efficiency of new and traditional energy sources.
2. Industrial restructuring needs to be accelerated and medium- and long-term development plans and energy plans need to be developed. In combination with the development and utilization of new technologies for traditional energy, we should actively promote the adjustment of energy structure and industrial structure. Traditional energy consumption plays and will continue to play an important role in urban development and economic growth for decades in the coming future. However, traditional energy consumption also brings problems such as increased CO_2_ emissions and air pollution, all of which affect sustainable development. Since China joined the Paris Climate Change Agreement on 3 September 2016, the Chinese government adopted a series of measures for domestic greenhouse gas emission reductions. The “13th Five-Year Plan” carbon intensity reduction target aims at controlling both total energy consumption and total energy intensity, strengthening low-carbon city pilot demonstrations, promoting the development of a national carbon trading market, and planning and implementing supporting policies and measures. At the same time, China is seeking to optimize its energy structure, with the proportion of coal being used for power generation dropping from 72% in 2005 to 64% in 2015, with a further drop to 60% expected by 2020. The empirical results suggested that increased CO_2_ emission reduction efforts are needed in Anhui, Jiangsu, Shandong, Shanxi, and Shaanxi, and further improvements are needed in Chongqing, Fujian, Gansu, Guangdong, Guangxi, Henan, Hubei, Hunan, Jiangxi, Qinghai, Sichuan, Xinjiang, Yunnan, and Zhejiang. NO_2_ emission reductions are needed in Guizhou and Liaoning, and all undesirable pollutant output indicators need to be improved in Jilin, Hebei, and Ningxia.
3. Most regions need to actively strengthen the source control of air pollutant emissions. They need to actively develop and adopt new technologies and clean energy technologies to control the air pollutants of high-polluting manufacturing enterprises at the source and discharge process. The current main governance measure is end-of-pipe governance, whereby once mandatory end-of-pipe governance is not strictly enforced, as emissions of air pollutants from companies that need to recover from economic development still exist. Therefore, effective measures should be to encourage enterprises to adopt new technologies and clean energy use technologies to establish green ecological enterprise production through the production process of enterprises, and fundamentally reduce air pollutant emissions in the long run.
4. Actively promoting the research, development, and utilization of clean renewable energy, and actively promoting the use of new energy in production are positive and effective measures to improve environmental efficiency. China’s renewable energy installed equipment capacity currently accounts for 16% to 20% of global capacity. Compared to traditional energy and other indicators, the new energy efficiencies in most municipalities/provinces were very low, except for Beijing, Inner Mongolia, Shanghai, and Tianjin, all of which had efficiencies of 1. Therefore, all municipalities/provinces need to put greater focus on new energy development and improving traditional energy efficiencies.
5. Comprehensive governance plans and measures need to be developed to jointly manage carbon dioxide emissions and air pollutant emissions. In most regions, carbon dioxide emissions in recent years not only have room for improvement, but efficiency scores also showed a downward trend. Emissions and inefficiencies in air pollutants exacerbate the pressure on environmental protection efforts. It is necessary to explore and actively promote measures and policies to jointly manage carbon dioxide emissions and air pollutant emissions.

## Figures and Tables

**Table 1:** Input and output variables. GDP—gross domestic product.

Input Variables	Output Variables	Undesirable Output
Labor (lab)	GDP	CO_2_
Fixed assets (asset)		SO_2_
Energy consumption (com)		NO_2_
New energy		

**Table 2:** Epsilon score. EBM—Epsilou-based measure.

Epsilon Score	2013	2014	2015	2016
Epsilon for EBM X	0.2427	0.3584	0.2698	0.2771
Epsilon for EBM Y	0.093	0.1450	0.1105	0.1264

**Table 3:** Efficiency in each municipality (m)/province from 2013–2016. DMU—decision-making unit.

No.	DMU	2013	2014	2015	2016
1	Anhui	0.6980	0.6680	0.6644	0.6454
2	Beijing (m)	1.0000	1.0000	1.0000	1.0000
3	Chongqing (m)	0.6835	0.6542	0.6598	0.6493
4	Fujian	0.8130	0.7918	0.7760	0.7518
5	Gansu	0.4946	0.4673	0.4371	0.4147
6	Guangdong	0.8528	0.8411	0.8361	0.8264
7	Guangxi	0.7044	0.6921	0.7027	0.7070
8	Guizhou	0.5354	0.5366	0.5480	0.5478
9	Hainan	0.8228	0.8065	0.7693	0.7388
10	Hebei	0.7858	0.7261	0.6992	0.6577
11	Heilongjiang	0.6258	0.6593	0.6398	0.6553
12	Henan	0.6165	0.5955	0.5758	0.5567
13	Hubei	0.7500	0.7311	0.7272	0.7076
14	Hunan	0.8177	0.8008	0.8039	0.7977
15	Jiangsu	0.8475	0.8092	0.8259	0.8043
16	Jiangxi	0.6562	0.6158	0.5926	0.5666
17	Jilin	0.7298	0.7040	0.6829	0.6606
18	Liaoning	0.7399	0.7233	0.7983	1.0000
19	Inner Mongolia	1.0000	1.0000	1.0000	1.0000
20	Ningxia	0.6280	0.5889	0.5818	0.5559
21	Qinghai	0.6083	0.5990	0.5960	0.5918
22	Shandong	0.8248	0.8070	0.7988	0.7808
23	Shanghai (m)	1.0000	1.0000	1.0000	1.0000
24	Shanxi	0.5380	0.5061	0.4759	0.4503
25	Shaanxi	0.6070	0.5875	0.5686	0.5513
26	Sichuan	0.7174	0.7130	0.7099	0.7201
27	Tianjin (m)	1.0000	1.0000	1.0000	1.0000
28	Xinjiang	0.5344	0.5124	0.4755	0.4546
29	Yunnan	0.5725	0.5743	0.5674	0.5677
30	Zhejiang	0.8457	0.8047	0.7992	0.7706

**Table 4:** 2013 input and output indicator radial and non-radial inefficiency scores.

No.	DMU	Score	Input Inefficiency	Input Radial Inefficiency	Input Non-Radial Inefficiency	Output Inefficiency	Output Radial Inefficiency	Output Non-Radial Inefficiency
1	Anhui	0.6980	0.1981	0.1268	0.0712	0.1489	0.1268	0.0221
2	Beijing	1.0000	0.0000	0.0000	0.0000	0.0000	0.0000	0.0000
3	Chongqing	0.6835	0.2046	0.1516	0.0530	0.1637	0.1516	0.0121
4	Fujian	0.8130	0.1294	0.0641	0.0653	0.0708	0.0641	0.0067
5	Gansu	0.4946	0.3484	0.2912	0.0572	0.3175	0.2912	0.0263
6	Guangdong	0.8528	0.1069	0.0430	0.0639	0.0473	0.0430	0.0043
7	Guangxi	0.7044	0.1889	0.1384	0.0506	0.1515	0.1384	0.0132
8	Guizhou	0.5354	0.3162	0.2412	0.0750	0.2772	0.2412	0.0360
9	Hainan	0.8228	0.1374	0.0348	0.1026	0.0484	0.0348	0.0136
10	Hebei	0.7858	0.1445	0.0462	0.0983	0.0888	0.0462	0.0426
11	Heilongjiang	0.6258	0.2462	0.1789	0.0673	0.2045	0.1789	0.0255
12	Henan	0.6165	0.2503	0.1930	0.0573	0.2161	0.1930	0.0231
13	Hubei	0.7500	0.1652	0.1033	0.0619	0.1130	0.1033	0.0097
14	Hunan	0.8177	0.1227	0.0621	0.0606	0.0728	0.0621	0.0107
15	Jiangsu	0.8475	0.1075	0.0474	0.0601	0.0531	0.0474	0.0057
16	Jiangxi	0.6563	0.2204	0.1739	0.0466	0.1879	0.1739	0.0140
17	Jilin	0.7298	0.1767	0.1023	0.0744	0.1281	0.1023	0.0258
18	Liaoning	0.7399	0.1723	0.0947	0.0777	0.1187	0.0947	0.0240
19	Inner Mongolia	1.0000	0.0000	0.0000	0.0000	0.0000	0.0000	0.0000
20	Ningxia	0.6280	0.2486	0.1443	0.1044	0.1964	0.1443	0.0521
21	Qinghai	0.6083	0.2613	0.1818	0.0795	0.2144	0.1818	0.0326
22	Shandong	0.8248	0.1180	0.0442	0.0738	0.0694	0.0442	0.0252
23	Shanghai	1.0000	0.0000	0.0000	0.0000	0.0000	0.0000	0.0000
24	Shanxi	0.5380	0.3143	0.2338	0.0806	0.2745	0.2338	0.0407
25	Shaanxi	0.6070	0.2603	0.1921	0.0682	0.2186	0.1921	0.0266
26	Sichuan	0.7174	0.1839	0.1250	0.0589	0.1376	0.1250	0.0126
27	Tianjin	1.0000	0.0000	0.0000	0.0000	0.0000	0.0000	0.0000
28	Xinjiang	0.5344	0.3161	0.2416	0.0745	0.2798	0.2416	0.0382
29	Yunnan	0.5725	0.2858	0.2247	0.0611	0.2476	0.2247	0.0229
30	Zhejiang	0.8457	0.1157	0.0365	0.0793	0.0457	0.0365	0.0092

**Table 5:** 2014 input and output indicator radial and non-radial inefficiency scores.

No.	DMU	Score	Input Inefficiency	Input Radial Inefficiency	Input Non-Radial Inefficiency	Output Inefficiency	Output Radial Inefficiency	Output Non-Radial Inefficiency
1	Anhui	0.6680	0.2206	0.1423	0.0783	0.1667	0.1423	0.0244
2	Beijing	1.0000	0.0000	0.0000	0.0000	0.0000	0.0000	0.0000
3	Chongqing	0.6542	0.2228	0.1765	0.0463	0.1881	0.1765	0.0117
4	Fujian	0.7918	0.1435	0.0737	0.0698	0.0817	0.0737	0.0080
5	Gansu	0.4673	0.3724	0.3142	0.0582	0.3431	0.3142	0.0289
6	Guangdong	0.8411	0.1119	0.0534	0.0585	0.0559	0.0534	0.0024
7	Guangxi	0.6921	0.1970	0.1471	0.0499	0.1601	0.1471	0.0130
8	Guizhou	0.5366	0.3149	0.2378	0.0771	0.2766	0.2378	0.0388
9	Hainan	0.8065	0.1467	0.0428	0.1039	0.0580	0.0428	0.0152
10	Hebei	0.7261	0.1798	0.0855	0.0943	0.1296	0.0855	0.0441
11	Heilongjiang	0.6593	0.2220	0.1416	0.0804	0.1801	0.1416	0.0384
12	Henan	0.5955	0.2652	0.2093	0.0559	0.2338	0.2093	0.0245
13	Hubei	0.7311	0.1775	0.1154	0.0621	0.1251	0.1154	0.0097
14	Hunan	0.8008	0.1330	0.0718	0.0611	0.0827	0.0718	0.0109
15	Jiangsu	0.8092	0.1312	0.0674	0.0637	0.0737	0.0674	0.0063
16	Jiangxi	0.6158	0.2494	0.2048	0.0446	0.2189	0.2048	0.0140
17	Jilin	0.7040	0.1928	0.1163	0.0765	0.1467	0.1163	0.0304
18	Liaoning	0.7233	0.1797	0.1058	0.0739	0.1341	0.1058	0.0283
19	Inner Mongolia	1.0000	0.0000	0.0000	0.0000	0.0000	0.0000	0.0000
20	Ningxia	0.5889	0.2767	0.1724	0.1043	0.2283	0.1724	0.0560
21	Qinghai	0.5990	0.2674	0.1870	0.0804	0.2230	0.1870	0.0360
22	Shandong	0.8070	0.1278	0.0504	0.0774	0.0808	0.0504	0.0304
23	Shanghai	1.0000	0.0000	0.0000	0.0000	0.0000	0.0000	0.0000
24	Shanxi	0.5061	0.3404	0.2585	0.0819	0.3032	0.2585	0.0446
25	Shaanxi	0.5875	0.2753	0.2034	0.0719	0.2336	0.2034	0.0302
26	Sichuan	0.7130	0.1863	0.1284	0.0578	0.1412	0.1284	0.0128
27	Tianjin	1.0000	0.0000	0.0000	0.0000	0.0000	0.0000	0.0000
28	Xinjiang	0.5124	0.3341	0.2568	0.0773	0.2994	0.2568	0.0426
29	Yunnan	0.5743	0.2834	0.2219	0.0615	0.2477	0.2219	0.0258
30	Zhejiang	0.8047	0.1317	0.0707	0.0611	0.0791	0.0707	0.0084

**Table 6:** 2015 input and output indicator radial and non-radial inefficiency scores.

No.	DMU	Score	Input Inefficiency	Input Radial Inefficiency	Input Non-Radial Inefficiency	Output Inefficiency	Output Radial Inefficiency	Output Non-Radial Inefficiency
1	Anhui	0.6644	0.2266	0.1342	0.0924	0.1642	0.1342	0.0300
2	Beijing	1.0000	0.0000	0.0000	0.0000	0.0000	0.0000	0.0000
3	Chongqing	0.6598	0.2209	0.1672	0.0537	0.1808	0.1672	0.0135
4	Fujian	0.7760	0.1559	0.0787	0.0772	0.0877	0.0787	0.0090
5	Gansu	0.4371	0.4014	0.3378	0.0636	0.3696	0.3378	0.0318
6	Guangdong	0.8361	0.1196	0.0493	0.0703	0.0531	0.0493	0.0038
7	Guangxi	0.7027	0.1936	0.1334	0.0602	0.1476	0.1334	0.0142
8	Guizhou	0.5480	0.3079	0.2215	0.0865	0.2629	0.2215	0.0414
9	Hainan	0.7693	0.1694	0.0635	0.1059	0.0797	0.0635	0.0162
10	Hebei	0.6992	0.1999	0.0956	0.1043	0.1442	0.0956	0.0485
11	Heilongjiang	0.6398	0.2374	0.1482	0.0893	0.1919	0.1482	0.0437
12	Henan	0.5758	0.2821	0.2202	0.0620	0.2467	0.2202	0.0265
13	Hubei	0.7272	0.1837	0.1094	0.0743	0.1225	0.1094	0.0131
14	Hunan	0.8039	0.1361	0.0585	0.0776	0.0747	0.0585	0.0162
15	Jiangsu	0.8259	0.1261	0.0476	0.0785	0.0580	0.0476	0.0104
16	Jiangxi	0.5926	0.2692	0.2154	0.0537	0.2333	0.2154	0.0179
17	Jilin	0.6829	0.2084	0.1241	0.0843	0.1592	0.1241	0.0351
18	Liaoning	0.7983	0.1347	0.0371	0.0976	0.0839	0.0371	0.0468
19	Inner Mongolia	1.0000	0.0000	0.0000	0.0000	0.0000	0.0000	0.0000
20	Ningxia	0.5818	0.2841	0.1697	0.1143	0.2305	0.1697	0.0607
21	Qinghai	0.5960	0.2707	0.1855	0.0851	0.2238	0.1855	0.0382
22	Shandong	0.7988	0.1356	0.0429	0.0927	0.0822	0.0429	0.0393
23	Shanghai	1.0000	0.0000	0.0000	0.0000	0.0000	0.0000	0.0000
24	Shanxi	0.4759	0.3685	0.2803	0.0882	0.3269	0.2803	0.0466
25	Shaanxi	0.5686	0.2920	0.2105	0.0815	0.2451	0.2105	0.0346
26	Sichuan	0.7099	0.1880	0.1325	0.0555	0.1439	0.1325	0.0114
27	Tianjin	1.0000	0.0000	0.0000	0.0000	0.0000	0.0000	0.0000
28	Xinjiang	0.4755	0.3681	0.2844	0.0837	0.3289	0.2844	0.0445
29	Yunnan	0.5674	0.2899	0.2250	0.0649	0.2516	0.2250	0.0266
30	Zhejiang	0.7992	0.1392	0.0651	0.0741	0.0772	0.0651	0.0121

**Table 7:** 2016 input and output indicator radial and non-radial inefficiency scores.

No.	DMU	Score	Input Inefficiency	Input Radial Inefficiency	Input Non-Radial Inefficiency	Output Inefficiency	Output Radial Inefficiency	Output Non-Radial Inefficiency
1	Anhui	0.6454	0.2397	0.1418	0.0979	0.1779	0.1418	0.0361
2	Beijing	1.0000	0.0000	0.0000	0.0000	0.0000	0.0000	0.0000
3	Chongqing	0.6493	0.2289	0.1682	0.0607	0.1876	0.1682	0.0194
4	Fujian	0.7518	0.1709	0.0925	0.0784	0.1027	0.0925	0.0102
5	Gansu	0.4147	0.4221	0.3566	0.0656	0.3935	0.3566	0.0370
6	Guangdong	0.8264	0.1224	0.0575	0.0649	0.0620	0.0575	0.0044
7	Guangxi	0.7070	0.1924	0.1252	0.0672	0.1423	0.1252	0.0171
8	Guizhou	0.5478	0.3075	0.2178	0.0897	0.2641	0.2178	0.0463
9	Hainan	0.7388	0.1876	0.0805	0.1071	0.0997	0.0805	0.0192
10	Hebei	0.6577	0.2266	0.1219	0.1047	0.1758	0.1219	0.0539
11	Heilongjiang	0.6553	0.2254	0.1241	0.1013	0.1821	0.1241	0.0580
12	Henan	0.5567	0.2970	0.2325	0.0646	0.2628	0.2325	0.0303
13	Hubei	0.7076	0.1962	0.1212	0.0750	0.1359	0.1212	0.0147
14	Hunan	0.7977	0.1405	0.0554	0.0852	0.0774	0.0554	0.0220
15	Jiangsu	0.8043	0.1399	0.0559	0.0840	0.0694	0.0559	0.0135
16	Jiangxi	0.5666	0.2899	0.2306	0.0593	0.2533	0.2306	0.0228
17	Jilin	0.6606	0.2226	0.1341	0.0885	0.1768	0.1341	0.0427
18	Liaoning	1.0000	0.0000	0.0000	0.0000	0.0000	0.0000	0.0000
19	Inner Mongolia	1.0000	0.0000	0.0000	0.0000	0.0000	0.0000	0.0000
20	Ningxia	0.5559	0.3022	0.1867	0.1155	0.2552	0.1867	0.0685
21	Qinghai	0.5918	0.2714	0.1883	0.0832	0.2311	0.1883	0.0428
22	Shandong	0.7808	0.1468	0.0441	0.1026	0.0928	0.0441	0.0487
23	Shanghai	1.0000	0.0000	0.0000	0.0000	0.0000	0.0000	0.0000
24	Shanxi	0.4503	0.3906	0.3006	0.0900	0.3533	0.3006	0.0527
25	Shaanxi	0.5513	0.3051	0.2193	0.0858	0.2603	0.2193	0.0410
26	Sichuan	0.7201	0.1812	0.1212	0.0600	0.1370	0.1212	0.0158
27	Tianjin	1.0000	0.0000	0.0000	0.0000	0.0000	0.0000	0.0000
28	Xinjiang	0.4546	0.3859	0.2996	0.0863	0.3507	0.2996	0.0511
29	Yunnan	0.5677	0.2880	0.2238	0.0642	0.2541	0.2238	0.0304
30	Zhejiang	0.7706	0.1574	0.0779	0.0795	0.0935	0.0779	0.0156

**Table 8:** 2013–2016 asset and employment (em) efficiencies.

DMU	2013 Assets	2014 Assets	2015 Assets	2016 Assets	2013 em	2014 em	2015 em	2016 em
Anhui	0.7356	0.7281	0.7322	0.7308	0.8732	0.8577	0.8660	0.7356
Beijing	1.0000	1.0000	1.0000	1.0000	1.0000	1.0000	1.0000	1.0000
Chongqing	0.7337	0.8235	0.8328	0.8318	0.8484	0.8235	0.8330	0.7337
Fujian	0.8294	0.8055	0.7898	0.7946	0.9359	0.9263	0.9210	0.8294
Gansu	0.7088	0.6858	0.6622	0.6434	0.7088	0.6858	0.6620	0.7088
Guangdong	0.7707	0.8714	0.8100	0.9425	0.9570	0.9466	0.9510	0.7707
Guangxi	0.8616	0.8529	0.8184	0.7839	0.8617	0.8529	0.8670	0.8616
Guizhou	0.7588	0.7622	0.7785	0.7822	0.7588	0.7622	0.7790	0.7588
Hainan	0.3228	0.3501	0.3933	0.4648	0.9652	0.9572	0.9370	0.3228
Hebei	0.8111	0.8008	0.7934	0.7840	0.9538	0.9145	0.9040	0.8111
Heilongjiang	0.8211	0.8584	0.8518	0.8759	0.8211	0.8584	0.8520	0.8211
Henan	0.8070	0.7907	0.7798	0.7675	0.8070	0.7907	0.7800	0.8070
Hubei	0.8492	0.8426	0.8283	0.8373	0.8967	0.8846	0.8910	0.8492
Hunan	0.9111	0.9017	0.8739	0.8588	0.9379	0.9282	0.9420	0.9111
Jiangsu	0.9526	0.9326	0.9524	0.9441	0.9526	0.9326	0.9520	0.9526
Jiangxi	0.7798	0.7952	0.7846	0.7694	0.8261	0.7952	0.7850	0.7798
Jilin	0.8977	0.8837	0.8759	0.8659	0.8977	0.8837	0.8760	0.8977
Liaoning	0.7501	0.8561	0.9629	1.0000	0.9053	0.8942	0.9630	0.7501
Inner Mongolia	1.0000	1.0000	1.0000	1.0000	1.0000	1.0000	1.0000	1.0000
Ningxia	0.7033	0.6799	0.6951	0.6890	0.8557	0.8276	0.8300	0.7033
Qinghai	0.6715	0.6391	0.6385	0.6246	0.8182	0.8130	0.8140	0.6715
Shandong	0.9558	0.9496	0.9571	0.9559	0.9558	0.9496	0.9570	0.9558
Shanghai	1.0000	1.0000	1.0000	1.0000	1.0000	1.0000	1.0000	1.0000
Shanxi	0.7662	0.7415	0.7197	0.6994	0.7663	0.7415	0.7200	0.7662
Shaanxi	0.8079	0.7966	0.7895	0.7807	0.8079	0.7966	0.7900	0.8079
Sichuan	0.8224	0.8306	0.8593	0.8462	0.8750	0.8716	0.8670	0.8224
Tianjin	1.0000	1.0000	1.0000	1.0000	1.0000	1.0000	1.0000	1.0000
Xinjiang	0.7584	0.7432	0.7156	0.7004	0.7584	0.7432	0.7160	0.7584
Yunnan	0.7753	0.7781	0.7750	0.7762	0.7753	0.7781	0.7750	0.7753
Zhejiang	0.6749	0.9293	0.9349	0.9221	0.9635	0.9293	0.9350	0.6749

**Table 9:** 2013–2016 new energy (New), energy (Con), and GDP efficiencies.

No.	DMU	2013 Con	2014 Con	2015 Con	2016 Con	2013 New	2014 New	2015 New	2016 New	2013 GDP	2014 GDP	2015 GDP	2016 GDP
1	Anhui	0.5056	0.4822	0.4371	0.4063	0.1842	0.0826	0.0488	0.0213	0.8988	0.8754	0.8942	0.8895
2	Beijing	1.0000	1.0000	1.0000	1.0000	1.0000	1.0000	1.0000	1.0000	1.0000	1.0000	1.0000	1.0000
3	Chongqing	0.8484	0.8235	0.7965	0.7282	0.0288	0.0247	0.0298	0.0289	0.8837	0.8500	0.8747	0.8741
4	Fujian	0.8252	0.7872	0.7786	0.7541	0.0167	0.0166	0.0135	0.0135	0.9432	0.9314	0.9320	0.9219
5	Gansu	0.4335	0.4034	0.3603	0.3267	0.0064	0.0063	0.0066	0.0069	0.8160	0.7609	0.7984	0.7919
6	Guangdong	0.9570	0.9466	0.9259	0.8891	0.0240	0.0218	0.0271	0.0190	0.9604	0.9493	0.9552	0.9484
7	Guangxi	0.8133	0.8369	0.8339	0.8257	0.0120	0.0102	0.0093	0.0100	0.8916	0.8718	0.8947	0.8999
8	Guizhou	0.2533	0.2632	0.2648	0.2644	0.0072	0.0058	0.0059	0.0060	0.8373	0.8079	0.8465	0.8483
9	Hainan	0.8132	0.7866	0.7543	0.7164	0.0358	0.0380	0.0731	0.0167	0.9675	0.9590	0.9437	0.9307
10	Hebei	0.3435	0.3445	0.3097	0.2988	0.0705	0.0696	0.0649	0.0459	0.9577	0.9212	0.9197	0.9020
11	Heilongjiang	0.4404	0.3430	0.3023	0.2441	0.0614	0.0644	0.0762	0.0536	0.8682	0.8759	0.8857	0.9006
12	Henan	0.5239	0.5132	0.4871	0.4652	0.1117	0.1451	0.1398	0.1298	0.8608	0.8269	0.8471	0.8413
13	Hubei	0.7540	0.7568	0.6987	0.6825	0.0082	0.0079	0.0089	0.0081	0.9144	0.8965	0.9102	0.9024
14	Hunan	0.8288	0.8356	0.7535	0.7112	0.0093	0.0096	0.0097	0.0099	0.9448	0.9330	0.9476	0.9502
15	Jiangsu	0.7549	0.7306	0.6613	0.6191	0.1075	0.0654	0.0502	0.0376	0.9567	0.9368	0.9565	0.9497
16	Jiangxi	0.8261	0.7851	0.7029	0.6347	0.0441	0.0382	0.0329	0.0224	0.8710	0.8300	0.8494	0.8422
17	Jilin	0.5012	0.4671	0.4390	0.4087	0.0296	0.0430	0.0495	0.0401	0.9151	0.8958	0.9006	0.8943
18	Liaoning	0.6005	0.5621	0.4275	1.0000	0.0477	0.0439	0.0394	1.0000	0.9204	0.9043	0.9655	1.0000
19	Inner Mongolia	1.0000	1.0000	1.0000	1.0000	1.0000	1.0000	1.0000	1.0000	1.0000	1.0000	1.0000	1.0000
20	Ningxia	0.1263	0.1164	0.1049	0.0953	0.0138	0.0106	0.0088	0.0066	0.8880	0.8530	0.8733	0.8641
21	Qinghai	0.4422	0.4811	0.5215	0.5908	0.0021	0.0022	0.0025	0.0027	0.8667	0.8425	0.8647	0.8632
22	Shandong	0.5497	0.4948	0.4371	0.3886	0.1094	0.1379	0.0937	0.0398	0.9594	0.9520	0.9605	0.9594
23	Shanghai	1.0000	1.0000	1.0000	1.0000	1.0000	1.0000	1.0000	1.0000	1.0000	1.0000	1.0000	1.0000
24	Shanxi	0.1449	0.1252	0.1130	0.0986	0.0505	0.0372	0.0257	0.0122	0.8407	0.7946	0.8204	0.8123
25	Shaanxi	0.4063	0.3623	0.3164	0.2786	0.0565	0.0558	0.0482	0.0458	0.8612	0.8310	0.8519	0.8476
26	Sichuan	0.7674	0.7975	0.8663	0.8788	0.0054	0.0049	0.0048	0.0049	0.9000	0.8862	0.8953	0.9024
27	Tianjin	1.0000	1.0000	1.0000	1.0000	1.0000	1.0000	1.0000	1.0000	1.0000	1.0000	1.0000	1.0000
28	Xinjiang	0.2546	0.2186	0.1804	0.1529	0.0130	0.0123	0.0109	0.0094	0.8371	0.7957	0.8187	0.8127
29	Yunnan	0.5002	0.5294	0.5580	0.5992	0.0030	0.0025	0.0027	0.0023	0.8450	0.8184	0.8448	0.8454
30	Zhejiang	0.7970	0.7703	0.6963	0.6405	0.0671	0.0590	0.0423	0.0353	0.9660	0.9340	0.9424	0.9326

**Table 10:** 2013–2016 CO_2_, SO_2_, and NO_2_ efficiencies.

No.	DMU	2013 CO_2_	2014 CO_2_	2015 CO_2_	2016 CO_2_	2013 SO_2_	2014 SO_2_	2015 SO_2_	2016 SO_2_	2013 NO_2_	2014 NO_2_	2015 NO_2_	2016 NO_2_
1	Anhui	0.5056	0.4822	0.4371	0.4063	0.6489	0.6426	0.5844	0.5584	0.5415	0.5295	0.5166	0.4991
2	Beijing	1.0000	1.0000	1.0000	1.0000	1.0000	1.0000	1.0000	1.0000	1.0000	1.0000	1.0000	1.0000
3	Chongqing	0.8484	0.8235	0.7965	0.7282	0.3622	0.3936	0.3932	0.3901	0.8270	0.8129	0.8303	0.7789
4	Fujian	0.8252	0.7872	0.7786	0.7541	0.7625	0.7611	0.7462	0.7434	0.9359	0.9263	0.9213	0.9075
5	Gansu	0.4335	0.4034	0.3603	0.3267	0.2052	0.1942	0.1671	0.1514	0.3848	0.3721	0.3385	0.3147
6	Guangdong	0.9570	0.9466	0.9259	0.8891	0.8190	0.8634	0.8403	0.8581	0.9151	0.9375	0.9507	0.9425
7	Guangxi	0.8133	0.8369	0.8666	0.8748	0.5192	0.5066	0.4923	0.4633	0.7010	0.7249	0.7413	0.7602
8	Guizhou	0.2533	0.2632	0.2648	0.2644	0.1423	0.1538	0.1617	0.1691	0.3738	0.4043	0.4467	0.4843
9	Hainan	0.8132	0.7866	0.7543	0.7164	0.9652	0.9572	0.9365	0.9195	0.5514	0.5580	0.5522	0.5359
10	Hebei	0.3435	0.3445	0.3097	0.2988	0.3478	0.3570	0.3312	0.3302	0.3887	0.3790	0.3608	0.3476
11	Heilongjiang	0.4404	0.3430	0.3023	0.2441	0.4847	0.4007	0.3484	0.2901	0.4691	0.3832	0.3640	0.3102
12	Henan	0.5239	0.5132	0.4871	0.4652	0.4245	0.4270	0.4058	0.3952	0.5065	0.5078	0.5110	0.5085
13	Hubei	0.7540	0.7568	0.6987	0.6825	0.6298	0.6431	0.6171	0.6186	0.8967	0.8846	0.8906	0.8788
14	Hunan	0.8288	0.8356	0.7535	0.7112	0.5961	0.6080	0.5775	0.5604	0.9379	0.9282	0.9187	0.8809
15	Jiangsu	0.7549	0.7306	0.6613	0.6191	0.9109	0.8950	0.8713	0.8493	0.9526	0.9326	0.9524	0.9441
16	Jiangxi	0.8261	0.7851	0.7029	0.6347	0.4388	0.4551	0.4163	0.4001	0.6270	0.6177	0.6040	0.5735
17	Jilin	0.5012	0.4671	0.4390	0.4087	0.5612	0.5408	0.4848	0.4546	0.5511	0.4988	0.4679	0.4211
18	Liaoning	0.6005	0.5621	0.4275	1.0000	0.4355	0.4234	0.3052	1.0000	0.6730	0.6300	0.5048	1.0000
19	Inner Mongolia	1.0000	1.0000	1.0000	1.0000	1.0000	1.0000	1.0000	1.0000	1.0000	1.0000	1.0000	1.0000
20	Ningxia	0.1263	0.1164	0.1049	0.0953	0.1136	0.1138	0.1071	0.1045	0.1455	0.1433	0.1383	0.1338
21	Qinghai	0.4422	0.4811	0.5215	0.5908	0.2403	0.2357	0.2136	0.2026	0.4093	0.3648	0.3628	0.3279
22	Shandong	0.5497	0.4948	0.3177	0.2624	0.5156	0.5109	0.4793	0.4634	0.7451	0.6951	0.6852	0.6395
23	Shanghai	1.0000	1.0000	1.0000	1.0000	1.0000	1.0000	1.0000	1.0000	1.0000	1.0000	1.0000	1.0000
24	Shanxi	0.1449	0.1252	0.1130	0.0986	0.1713	0.1600	0.1505	0.1410	0.2776	0.2558	0.2513	0.2364
25	Shaanxi	0.4063	0.3623	0.3164	0.2786	0.3566	0.3537	0.3214	0.3083	0.8877	0.5343	0.5094	0.4901
26	Sichuan	0.7674	0.7975	0.8663	0.8788	0.4526	0.4627	0.4675	0.4529	0.8750	0.8716	0.8675	0.8187
27	Tianjin	1.0000	1.0000	1.0000	1.0000	1.0000	1.0000	1.0000	1.0000	1.0000	1.0000	1.0000	1.0000
28	Xinjiang	0.2546	0.2186	0.1804	0.1529	0.1771	0.1701	0.1627	0.1534	0.2456	0.2342	0.2355	0.2242
29	Yunnan	0.5002	0.5294	0.5580	0.5992	0.2985	0.2963	0.2970	0.2940	0.5669	0.5351	0.5331	0.5025
30	Zhejiang	0.7970	0.7703	0.6962	0.6405	0.7425	0.7696	0.7441	0.7193	0.9635	0.9293	0.9349	0.9221

**Table 11:** Impact and analysis of the contaminant efficiencies.

DMU		CO_2_	SO_2_	NO_2_
Anhui	All three undesirable outputs had low efficiency scores. The efficiency of CO_2_ was relatively low and showed a downward trend and should be treated first.	▲		
Chongqing	SO_2_ had the lowest efficiency; NO_2_ had the best efficiency; therefore, more effective measures are needed to reduce SO_2_ emissions.		▲	
Fujian	NO_2_ had the best efficiency; the SO_2_ efficiency was lower than the others; therefore, more effective measures are needed to reduce SO_2_ emissions.		▲	
Gansu	All three emission indicators had poor efficiency scores, but SO2 had the lowest efficiency, and the CO_2_ efficiency was better; therefore, more effective measures are needed to reduce SO_2_ emissions.		▲	
Guangdong	SO_2_ efficiency was lower than the others; therefore, more effective measures are needed to reduce SO_2_ emissions.		▲	
Guangxi	SO_2_ efficiency was lower than the others; therefore, more effective measures are needed to reduce SO_2_ emissions.		▲	
Guizhou	All three undesirable outputs had low efficiencies below 0.4, with the SO_2_ efficiency being the lowest at below 0.2. Comprehensive management should be strengthened, after strengthening the governance of SO_2_ emissions.		▲	
Hainan	NO_2_ had the worst emission efficiency, The SO_2_ efficiency was high, but declined; thus, more effective measures are needed to reduce NO_2_ emissions.			▲
Hebei	All three undesirable indicator efficiencies were lower than 0.4, with the NO_2_ efficiency being slightly higher than CO_2_ and SO_2_. Comprehensive management should be strengthened, after strengthening the governance of CO_2_ emissions.	▲	▲	▲
Heilongjiang	All three undesirable indicator efficiencies were lower than 0.4, with the NO_2_ efficiency being slightly higher than CO_2_ and SO_2_. Comprehensive management should be strengthened, after strengthening the governance of CO_2_ emissions.	▲	▲	
Henan	The SO_2_ efficiency was the worst at only 0.4, and the CO_2_ efficiency score was between 0.4 and 0.6 but declined. The NO_2_ efficiency was similar to SO_2_, but with less fluctuation; Overall, more effective measures are needed to reduce SO_2_, CO_2_, and NO_2_ emissions, but the governance of SO_2_ emissions should be strengthened first.		▲	
Hubei	The NO_2_ efficiency was the highest at over 0.8, the CO_2_ efficiency was slightly lower, and the SO_2_ efficiency score was much lower; therefore, more effective measures are needed to reduce SO_2_ emissions.		▲	
Hunan	The SO_2_ efficiency score was much lower at 0.6; therefore, more effective measures are needed to reduce SO_2_ emissions.		▲	
Jiangsu	The CO_2_ efficiency was much lower than the other indicators at between 0.6 and 0.75; the reduction of CO_2_ emissions should become the focus of governance in Jiangsu.	▲		
Jiangxi	SO_2_ had the lowest efficiency at between 0.4 and 0.5 and declined; therefore, more effective measures are needed to reduce SO_2_ emissions. NO_2_ also declined at 0.6, with the CO_2_ efficiency being slightly better, declining to between 0.6 and 0.8; therefore, there is room for improvement.		▲	
Jilin	The NO_2_, SO_2_, and CO_2_ efficiencies were all between 0.6 and 0.8; therefore, the room for improvement was similar and these emissions should be treated equally. CO_2_ efficiency was slightly lower than others, and can be prioritized.	▲	▲	▲
Liaoning	The NO_2_, SO_2_, and CO_2_ efficiencies were similar, maintaining a continuous upward trend and reaching 1 in 2016. The state of input and output should be maintained in 2016, and attention should be paid to and sufficient measures should be taken to maintain the existing input and output status.	▲	▲	▲
Ningxia	All NO_2_, SO_2_, and CO_2_ efficiencies were very low at between 0.1 and 0.2, and declined; therefore, all three indicators need to improve. CO_2_ and SO_2_ emission efficiency were lower and can be prioritized.	▲	▲	▲
Qinghai	The SO_2_ efficiency was below 0.2 and had a downward trend. The CO_2_ efficiency was slightly higher at between 0.4 and 0.6, and the NO_2_ efficiency was between 0.2 and 0.4; therefore, all three indicators need to improve. The governance of SO_2_ emissions should be strengthened first.		▲	
Shandong	The CO_2_ efficiency score was between 0.2 and 0.8 and declined. The SO_2_ efficiency also declined; however, the minimum value of 0.4 was slightly better than the CO_2_ minimum. The efficiency of CO_2_ dropped sharply and should be treated first.	▲		
Shanxi	All NO_2_, SO_2_, and CO_2_ efficiencies were very low, with the CO_2_ efficiency being the lowest at 0.2 and continuing to decline. The SO_2_ efficiency was slightly better at around 0.2, but also decreased, and the NO_2_ efficiency was slightly better, but was only 0.3; therefore, all three indicators need to improve.	▲	▲	
Shaanxi	The CO_2_ efficiency was between 0.2 and 0.4, and declined. The SO_2_ efficiency also declined, with the minimum value being between 0.2 and 0.4. The NO_2_ efficiency was between 0.4 and 0.6, but also showed a downward trend; therefore, all three indicators need to improve.	▲	▲	
Sichuan	The SO_2_ efficiency was 0.5, and the CO_2_ efficiency was 0.8 and rising. The NO_2_ had a higher rising efficiency score between 0.8 and 0.9; therefore, more effective measures are needed to reduce SO_2_ emissions.		▲	
Xinjiang	All NO_2_, SO_2_, and CO_2_ efficiencies were very low. The SO_2_ efficiency score of 0.2 was the lowest and declined. The SO_2_ efficiency score was the worst overall and should be prioritized. However, the emission efficiency of the other two indicators was not high, and comprehensive management is also needed.		▲	
Yunnan	SO_2_ had the lowest efficiency at around 0.3 and declined, while the CO_2_ efficiency was better between 0.5 and 0.8 and rising. The NO_2_ efficiency at 0.5 to 0.6 was falling; therefore, more effective measures are needed to reduce SO_2_ emissions.		▲	
Zhejiang	The CO_2_ and SO_2_ efficiencies were between 0.6 and 0.8 and falling; therefore, more effective measures are needed to reduce CO_2_ and SO_2_ emissions.	▲	▲	

## References

[1] Hu J.L., Wang S.C. (2006). Total-factor energy efficiency of regions in China. Energy Policy.

[2] Yeh T.-L., Chen T.-Y., Lai P.-Y. (2010). A comparative study of energy utilization efficiency between Taiwan and China. Energy Policy.

[3] Shi G.-M., Bi J., Wang J.-N. (2010). Chinese regional industrial energy efficiency evaluation based on a DEA model of fixing non-energy inputs. Energy Policy.

[4] Choi Y., Zhang N., Zhou P. (2012). Efficiency and abatement costs of energy-related CO_2_ emissions in China: A slacks-based efficiency measure. Appl. Energy.

[5] Wu A.-H., Cao Y.-Y., Liu B. (2014). Energy efficiency evaluation for regions in China: An application of DEA and Malmquist indices. Energy Effic..

[6] Chang M.-C. (2014). Energy intensity, target level of energy intensity, and room for improvement in energy intensity: An application to the study of regions in the EU. Energy Policy.

[7] Wang K., Wei Y.-M. (2014). China’s regional industrial energy efficiency and carbon emissions abatement costs. Appl. Energy.

[8] Cui Q., Kuang H.-B., Wu C.-Y., Li Y. (2014). The changing trend and influencing factors of energy efficiency: The case of nine countries. Energy.

[9] Wu J., Lv L., Sun J. (2015). A comprehensive analysis of China’s regional energy saving and emission reduction efficiency: From production and treatment perspectives. Energy Policy.

[10] Pang R.-Z., Deng Z.-Q., Hu J.-L. (2015). Clean energy use and total-factor efficiencies: An international comparison. Renew. Sustain. Energy Rev..

[11] Guo X., Lu C.-C., Lee J.-H., Chiu Y.-H. (2017). Applying the dynamic DEA model to evaluate the energy efficiency of OECD countries and China Energy. Energy.

[12] Feng C., Zhang H., Huang J.-B. (2017). The Approach to realizing the potential of emissions reduction in China: An implication from data envelopment analysis. Renew. Sustain. Energy Rev..

[13] Chien T., Hu J.-L. (2007). Renewable energy and macroeconomic efficiency of OECD and non-OECD economies. Energy Policy.

[14] Honma S., Hu J.-L. (2008). Total-factor energy efficiency of regions in Japan. Energy Policy.

[15] Hoang V.-N., Rao D.S.P. (2010). Measuring and decomposing sustainable efficiency in agricultural production: A cumulative exergy balance approach. Ecol. Econ..

[16] Shiau T.-A., Jhang J.-S. (2010). An integration model of DEA and RST for measuring transport sustainability. Int. J. Sustain. Dev. World Econ..

[17] Blokhuis E., Advokaat B., Schaefer W. (2012). Assessing the performance of Dutch local energy companies. Energy Policy.

[18] Boubaker K. (2012). A review on renewable energy conceptual perspectives in North Africa using a polynomial optimization scheme. Renew. Sustain. Energy Rev..

[19] Menegaki A.N., Gurluk S. (2013). Greece and Turkey: Assessment and Comparison of Their Renewable Energy Performance. Int. J. Energy Econ. Policy.

[20] Fagiani R., Barquin J., Hakvoort R. (2013). Risk-Based Assessment of the Cost-Efficiency and the Effectivity of Renewable Energy Support Schemes: Certificate Markets versus Feed-In Tariffs. Energy Policy.

[21] Sueyoshi T., Goto M., Sugiyama M. (2013). DEA window analysis for environmental assessment in a dynamic time shift: Performance assessment of U.S. coal-fired power plants. Energy Econ..

[22] Sueyoshi T., Goto M. (2014). Environmental assessment for corporate sustainability by resource utilization and technology innovation: DEA radial measurement on Japanese industrial sectors. Energy Econ..

[23] Azlina A.A., Law S.H., Mustapha N.H.N. (2014). Dynamic linkages among transport energy consumption, income and CO_2_ emission in Malaysia. Energy Policy.

[24] de Castro Camioto F., Mariano E.B., do Nascimento Rebelatto D.A. (2014). Efficiency in Brazil’s industrial sectors in terms of energy and sustainable development. Environ. Sci. Policy.

[25] Wang H. (2015). A generalized MCDA–DEA (multi-criterion decision analysis–data envelopment analysis) approach to construct slacks-based composite indicator. Energy.

[26] Kim K.-T., Lee D.J., Park S.-J., Zhang Y., Sultanov A. (2015). Measuring the efficiency of the investment for renewable energy in Korea using data envelopment analysis. Renew. Sustain. Energy Rev.

[27] Zhang N., Xie H. (2015). Toward green IT: Modeling sustainable production characteristics for Chinese electronic information industry, 1980–2012. Technol. Forecast. Soc. Chang..

[28] Guo X., Zhu Q., Lv L., Chu J., Wu J. (2017). Efficiency evaluation of regional energy saving and emission reduction in China: A modified slacks-based measure approach. J. Clean. Prod..

[29] Tone K., Tsutsui M. (2010). Dynamic DEA: A Slacks-based Measure Approach. Omega.

[30] Zhu J. (2014). Quantitative Models for Performance Evaluation and Benchmarking: Data Envelopment Analysis with Spreadsheets.

[31] Inman O.L., Anderson T.R., Harmon R.R. (2006). Predicting US jet fighter aircraft introductions from 1944 to 1982: a dogfight between regression and TFDEA. Technol. Forecast. Soc. Chang..

[32] Mardani A., Zavadskas E.K., Streimikiene D., Jusoh A., Khoshnoudi M. (2017). A Comprehensive review of data envelopment analysis (DEA) approach in energy efficiency. Renew. Sustain. Energy Rev..

[33] Cook D., Zhu J. (2005). Modeling Performance Measurement Applications and Implementation Issues in DEA.

[34] Martínez-Molina A., Tort-Ausina I., Cho S., Vivancos J.-L. (2016). Energy efficiency and thermal comfort in historic buildings: A review. Renew. Sustain. Energy Rev..

[35] Moya D., Torres R., Stegen S. (2016). Analysis of the Ecuadorian energy audit practices: A review of energy efficiency promotion. Renew. Sustain. Energy Rev..

[36] Bian Y., Hu M., Wang Y., Xu H. (2016). Energy efficiency analysis of the economic system in China during 1986–2012: A parallel slacks-based measure approach. Renew. Sustain. Energy Rev..

[37] Balitskiy S., Bilan Y., Strielkowski W., Štreimikienė D. (2016). Energy efficiency and natural gas consumption in the context of economic development in the European Union. Renew. Sustain. Energy Rev..

[38] Chandel S.S., Sharma A., Marwaha B.M. (2016). Review of energy efficiency initiatives and regulations for residential buildings in India. Renew. Sustain. Energy Rev..

[39] Apergis N., Payne J.E. (2010). Renewable energy consumption and economic growth: Evidence from a panel of OECD countries. Energy Policy.

[40] Menegaki A.N. (2011). Growth and renewable energy in Europe: A random effect model with evidence for neutrality hypothesis. Energy Econ..

[41] Bildirici M. (2012). The relationship between economic growth and energy consumption. Renew. Sustain. Energy Rev..

[42] Apergis N., Payne J.E. (2014). Renewable energy, Output, CO_2_ emission and fossil fuel prices in Central America: Evidence from a non-linear Panel Smooth transition vector error correction model. Energy Econ..

[43] Solarin S.A., Ozturk I. (2015). On the causal dynamics between hydroelectricity consumption and economic growth in Latin America countries. Renew. Sustain. Energy Rev..

[44] Chang T., Gupta R., Inglesi-Lotz R., Simo-Kengne B., Smithers D., Trembling A. (2015). Renewable energy and growth: Evidence from heterogeneous panel of G7countries using Granger causality. Renew. Sustain. Energy Rev..

[45] Ozbugday F.C., Erbas B.C. (2015). How effective are energy efficiency and renewable energy in curbing CO_2_ emissions in the long run? A heterogeneous panel data analysis. Energy.

[46] Jaforullah M., King A. (2015). Does the use of renewable energy sources mitigate CO_2_ emissions? A reassessment of the US evidence. Energy Econ..

[47] Bilgili F., Koçak E., Bulut U. (2016). The dynamic impact of renewable energy consumption on CO_2_ emissions: A revisited environmental Kuznets curve approach. Renew. Sustain. Energy Rev..

[48] Farrell M.J. (1957). The Measurement of Productive Efficiency. J. R. Stat. Soc..

[49] Charnes A., Cooper W.W., Rhodes E. (1978). Measuring the Efficiency of Decision Making Units. Eur. J. Oper. Res..

[50] Banker R.D., Charnes A., Cooper W.W. (1984). Some Models for Estimating Technical and Scale Inefficiencies in Data Envelopment Analysis. Manag. Sci..

[51] Tone K. (2001). A Slacks-based Measure of Efficiency in Data Envelopment Analysis. Eur. J. Oper. Res..

[52] National Bureau of Statistics of China (2017). China Statistical Yearbook. http://www.stats.gov.cn/.

[53] China Statistical Yearbooks Database (2017). Demographics and the Employment Statistical Yearbook of China, and the Statistical Yearbooks of All Cities.

[54] (2017). China’s Environmental and Protection Bureau Reports.

[55] (2012). China’s Energy Policy 2012.

